# Chromosome Analysis and Sorting Using Conventional Flow Cytometers

**DOI:** 10.1002/cpz1.718

**Published:** 2023-03-15

**Authors:** Bee Ling Ng

**Affiliations:** ^1^ Wellcome Sanger Institute, Cytometry Core Facility Hinxton Cambridge United Kingdom

**Keywords:** chromosomes, DAPI, flow karyotyping, propidium iodide

## Abstract

The fluorescent dyes Hoechst (HO) and Chromomycin A3 (CA3) are commonly used for bivariate flow karyotyping to distinguish individual chromosomes from one another based on differences in base composition and DNA content. However, analysis of chromosomes using this fluorescent dye combination requires a flow cytometer equipped with lasers of specific wavelengths and higher power than is typical of conventional flow cytometers. This unit presents a chromosome staining technique with a dye combination of DAPI and propidium iodide (PI). Chromosomes stained using this dye combination can be analyzed on conventional flow cytometers equipped with a typical configuration of lasers and optics. © 2023 The Authors. Current Protocols published by Wiley Periodicals LLC.

**Basic Protocol 1**: Cell culture and metaphase harvest of suspension cell line

**Alternate Protocol 1**: Cell culture and metaphase harvest of adherent cell line

**Basic Protocol 2**: Preparation of chromosome suspension using polyamine isolation buffer

**Basic Protocol 3**: Staining chromosomes with DAPI and propidium iodide

**Alternate Protocol 2**: Staining chromosomes with Hoechst and Chromomycin A3

**Basic Protocol 4**: Bivariate flow karyotyping on a cell analyzer

**Basic Protocol 5**: Bivariate flow karyotyping on a cell sorter

**Basic Protocol 6**: Purification of flow‐sorted chromosomes

## INTRODUCTION

Flow cytometric analysis of chromosomes, also known as flow karyotyping, has been a useful tool for the classification of chromosomes and the detection of numerical and structural changes in chromosome aberrations. The process of chromosome analysis using a flow cytometer involves the isolation of chromosomes from mitotic cells followed by staining with a pair of base‐specific fluorescent dyes. The use of the DNA dyes Hoechst (HO) and Chromomycin A3 (CA3) has been the preferred staining method in laboratories carrying out bivariate flow karyotyping. The most complete flow cytometric resolution of the human karyotype can be achieved using this dye combination (Arndt‐Jovin and Jovin, 1990). The bisbenzimidazole dye HO has a binding preference for adenine‐thymine (AT) rich sequences (Kubista, Akerman, and Nordén, 1987; Latt, 1975), while the anthraquinone‐based antibiotic CA3 has a binding preference for guanine‐cytosine (GC)‐rich sequences (Behr, Honikel, and Hartmann, 1969). These DNA stains provide information on the DNA base composition (the ratio of AT and GC base pairs) and the DNA content of the chromosomes. To obtain high‐resolution flow karyotypes, this dye combination requires the use of lasers set in the UV (330‐360 nm) and indigo range (440‐460 nm) at relatively high‐power settings. There has been some success in the flow cytometric analysis of chromosomes stained with HO and CA3 using low‐power air‐cooled lasers (Frey, Houck, Shenker, and Hoffman, 1994; Jia et al., 2016), but the application of flow karyotyping remains limited to laboratories with flow cytometers that are equipped with lasers at these wavelengths. Lasers operating at such wavelength ranges are usually an expensive custom option. Thus, most flow analyzers and sorters are equipped with the standard factory optical configuration.

To overcome this limitation, an alternative chromosome staining method has been developed (Ng, Fu, Graham, Hall, and Thompson, 2019). The protocol described here makes use of a fluorescence dye combination of DAPI and propidium iodide (PI) for chromosome staining. The staining method is easy‐to‐perform and is compatible with most mammalian cell culture. Flow karyotypes with well‐resolved chromosome peaks can be acquired using this dye combination on flow cytometers equipped with typical lasers and optical configuration.

## Basic Protocol 1 CELL CULTURE AND METAPHASE HARVEST OF SUSPENSION CELL LINE

This protocol employs a human lymphoblastoid cell line, GM7016A. Suspension cell lines are sub‐cultured to 50% with 50 ml fresh growth medium and grown for 24 hr before treatment with mitotic inhibitors, i.e., colcemid or demecolcine. This treatment results in mitotic arrest, which helps to accumulate the percentage of cells at metaphase.

### Materials

Human lymphoblastoid cell lines, GM7016A
RPMI 1640 medium with HEPES (Gibco, cat. no. 52400‐025) supplemented with 15% FBS (Gibco, cat. no. 10270106) and antibiotics (Sigma, cat. no. G6784)
10 µg/ml KaryoMAX Colcemid (Gibco, cat. no. 15212‐012) or 10 µg/ml Demecolcine (Sigma, cat. no. D1925)

T‐75 cm^2^ sterile culture flasks (StemCell Technologies, cat. no. 200‐0501)
50‐ml polypropylene centrifuge tube (Greiner Bio‐one, cat no. 346370)
2‐ml Pasteur pipette (Greiner Bio‐one, cat no. 612398)
Benchtop centrifuge (Eppendorf or equivalent)
Cell culture incubator (Panasonic or equivalent)
Fluorescence microscope with 10× to 20× objective and PI filter set (Zeiss or equivalent)

**Figure 1:**
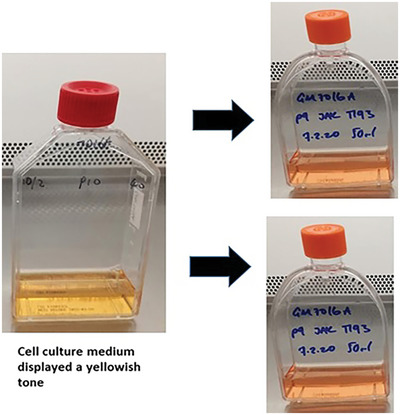
The near‐confluent cell culture medium will turn yellow, indicating that the cell culture needs feeding and sub‐culturing. In this example, the near‐confluent cells are sub‐cultured at a ratio of 1:1 in two separate flasks. The flasks are topped up with fresh medium, which is pink.

1 Culture cells in T‐75 cm^2^ flasks with 50 ml RPMI 1640 cell culture medium at 37°C in a 5% CO_2_ incubator.
2 The culture medium contains phenol red. When the medium turns yellow (see Fig. 1), which is an indication that the cells need subculturing, remove half of the spent culture medium without disturbing the cell clusters and subculture the near‐confluent cells at a ratio of 1:1 in two flasks.

**Figure 2:**
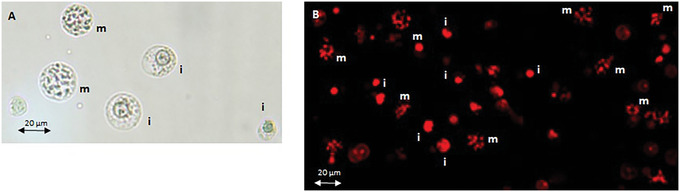
Images of chromosome preparation. Swollen cells in metaphase (m) and interphase (i) stained with (**A**) Turck's solution and (**B**) PI. i, cells in interphase; m cells in metaphase.

3 Bring the volume in the flasks to 50 ml with fresh culture medium. Incubate at 37°C in a 5% CO_2_ incubator.
4 After 24 hr, add colcemid to a final concentration of 0.1 µg/ml and then gently swirl the flask to mix well. Incubate for 6 hr.
  The duration of incubation with the mitotic inhibitors varies according to the rate of cell growth. A high mitotic index can be achieved by optimizing treatment time for each cell type. The number of cells at metaphase can be assessed during cell swelling with hypotonic solution (see Basic Protocol 2 and Fig. 2).
5 Collect cells suspended in the supernatant from both flasks (T‐75 cm^2^), transfer them to two 50‐ml polypropylene centrifuge tubes, and centrifuge at 289 × *g* for 5 min at room temperature.
6 Carefully remove the supernatant without disturbing the pellet. Invert the tube on an absorbent paper and drain the excess medium.
7 Proceed to Basic Protocol 2.

## Alternate Protocol 1 CELL CULTURE AND METAPHASE HARVEST OF ADHERENT CELL LINE

This modification of Basic Protocol 1 applies to cell culture and metaphase harvest for adherent cell lines. The protocol made use of a swine fibroblast cell line that should be applicable to other adherent cell lines.

### Additional Materials (also see Basic Protocol 1)

Swine fibroblast cell line (Sanger Source)
DMEM/F12 medium (Gibco, cat. no. 11330057) supplemented with 15% FBS (Gibco, cat. no. 10270106) and antibiotics mixture (Sigma, cat. no. G6784)
Sterile 1× PBS (Gibco, cat. no. 14190094)
TrypLE Express (Gibco, cat. no. 12604013)

T‐150 cm^2^ sterile culture flasks (StemCell Technologies, cat. no. 38072)
Inverted light microscope with 10× or 20× objective (Zeiss or equivalent)

1 Culture cells in T‐150 cm^2^ flasks with 25 ml DMEM at 37°C in a 5% CO_2_ incubator.
2 When the culture is near‐confluent (∼75% surface coverage), remove the spent medium.
3 Briefly rinse the cell layer with 5 ml sterile 1× PBS and then discard the buffer using a sterile 10‐ml pipette.
4 Add 3 ml TrypLE Express and ensure it covers the entire surface of the flask. Incubate at 37°C for 3‐5 min.
5 Tap the bottom of the flask gently to detach all of the cells. Add 5 ml fresh culture medium and mix well to obtain a single‐cell suspension.
6 Subculture the cell suspension at a ratio of 1:3 in four flasks. Bring the volume to 25 ml with fresh culture medium. Incubate at 37°C in a 5% CO_2_ incubator.
  In this example, a total of 8 flasks are used during the chromosome harvesting process. The number of flasks required to obtain a sufficient number of chromosomes (at least 5 million chromosomes per ml) will vary according to the total number of metaphase cells achieved after colcemid treatment.
7 After 24 hr, add colcemid to a final concentration of 0.1 µg/ml and gently swirl the flask to mix well. Incubate for 6‐16 hr.
  The duration of treatment with mitotic inhibitors varies according to the rate of cell growth. A high mitotic index can be achieved by optimizing treatment time for different cell types. Cells arrested at metaphase appear under the microscope as “rounded cells” loosely attached to the monolayer surface.
8 Gently tap the bottom of the flasks to dislodge the cells at metaphase.
9 Collect cells suspended in the supernatant from the four flasks (150 cm^2^) after mitotic shake‐off and transfer them to two 50‐ml polypropylene centrifuge tubes. Centrifuge at 289 × *g* for 5 min at room temperature.
10 Remove the supernatant without disturbing the pellet. Invert the tube on absorbent paper and drain the excess medium.
11 Proceed to Basic Protocol 2.

## Basic Protocol 2 PREPARATION OF CHROMOSOME SUSPENSION USING POLYAMINE ISOLATION BUFFER

In this protocol, the harvested colcemid‐treated cells are treated with hypotonic solution. The cells swell, increasing in volume, and the chromosomes spread apart within the plasma membrane. The cells are then disrupted mechanically to release the chromosomes after treatment with a modified polyamine isolation buffer (Ng and Carter, 2006).

### Materials

Hypotonic solution (see recipe)
Polyamine isolation buffer (PAB, see recipe)
Turck's stain (see recipe)
1 mg/ml propidium iodide (Sigma, cat. no. P4170) (see recipe)

50‐ml polypropylene centrifuge tube (Greiner Bio‐one, cat no. 346370)
15‐ml polypropylene centrifuge tube (Greiner Bio‐one, cat no. 188171)
5‐ml BD Falcon tube (SLS, cat no. 352054)
20‐µm mesh filter (CellTrics, cat. no. 04‐004‐2325)
2 ml Pasteur pipette (Greiner Bio‐one, cat. no. 612398)
Vortex (Fisherbrand or equivalent)

1 Resuspend the cell pellets from Basic Protocol 1 in hypotonic solution (∼5 ml per tube) with a Pasteur pipette. Combine the cell suspensions in a single 50‐ml polypropylene centrifuge tube and incubate at room temperature for about 15 min.
2 Mix 5 µl cell suspension with 5 µl Turck's stain on a microscope slide and directly monitor the cells for swelling under a light microscope.
  If Turck's stain is not available, use 1 mg/ml PI and view under a fluorescence microscope.
  Swollen cells at metaphase should display a good spread of chromosomes within the plasma membrane (see Fig. 2). Assess the cells for swelling and incubate for an additional 10 to 30 min if needed.

**Figure 3:**
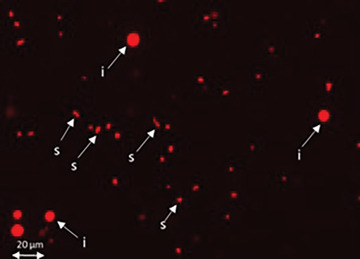
Images of chromosome preparation. Released single intact chromosomes (s) and interphase nuclei (i) after treating the swollen cells with PAB containing 0.25% (v/v) Triton X‐100, and staining with PI.

3 Centrifuge the swollen cells at 289 × *g* for 5 min at room temperature.
4 Remove the supernatant without disturbing the pellet. Invert the tube on absorbent paper and drain the excess medium.
5 Add 1 to 2 ml ice‐cold PAB to the cell pellet and gently resuspend using a Pasteur pipette. Incubate on ice for 10 min.
6 Disrupt the cells by vortexing for 10‐20 s.
7 Stain 5 µl suspension with 5 µl PI on a microscope slide and gently cover with a coverslip. View the stained chromosome suspension directly under the fluorescence microscope and check for single intact chromosomes (see Fig. 3).
  Continue to vortex as needed if numerous chromosome clumps (chromosomes that bundle to each other) are observed in the stained suspension. Alternatively, draw up and expel the suspension several times using a 5‐ml syringe attached to a 22.5 G needle.

8 Transfer the chromosome suspension to a 15‐ml polypropylene centrifuge tube and spin at 201 × *g* for 2 min at room temperature. Collect the supernatant in a 5‐ml Falcon tube after filtering through a 20‐µm mesh filter.
9 Proceed to Basic Protocol 3.

## Basic Protocol 3 STAINING CHROMOSOMES WITH DAPI AND PROPIDIUM IODIDE

The aim of this protocol is staining the chromosomes using DAPI and PI. This staining protocol is suitable for most conventional flow cytometers equipped with standard solid‐state lasers and optics configuration.

### Materials

1 mg/ml DAPI (Sigma, cat. no. D9542)
1 mg/ml PI (Sigma, cat. no. P4170)
1 M MgSO_4_·7H_2_O (Sigma, cat. no. M2773)
500 mM sodium sulfite (Sigma, cat. no. S8018)
1 M sodium citrate (Sigma, cat. no. S4641)

1 Per 1 ml chromosome suspension, stain overnight with 5 µl of 1 mg/ml DAPI (final concentration 5 µg/ml), 5 µl of 1 mg/ml PI (final concentration 5 µg/ml), 10 µl of 1 M MgSO_4_·7H_2_O (final concentration 10 mM), 10 µl sodium citrate (final concentration 10 mM), and 50 µl of 500 mM sodium sulfite (final concentration 25 mM). Store in the dark at 4°C overnight before flow analysis and sorting.
  Sodium citrate and sodium sulfite have been used to improve flow karyotype resolution for most lymphoblastoid cell lines. The effect of both compounds on resolution can vary by cell line. Monitor the flow karyotype by reducing the concentration or excluding one or the other compound.
2 Proceed to Basic Protocol 4 or Basic Protocol 5.

## Alternate Protocol 2 STAINING CHROMOSOMES WITH HOECHST AND CHROMOMYCIN A3

In this protocol, the chromosomes are stained using the conventional dye combination HO with CA3. This staining protocol is suitable for flow cytometers equipped with a laser wavelength set in the UV range (330‐360 nm) and another in the indigo range (440‐460 nm). Fluorescence detection of HO and CA3 is measured using a band‐pass BP 450/50 filter and a long‐pass LP 420 filter, respectively.

### Materials

1 mg/ml Hoechst 33258 (Sigma, cat. no. B2883)
10 mg/ml Chromomycin A3 (Sigma, cat. no. C2659)
1 M MgSO_4_·7H_2_O (Sigma, cat. no. M2773)
1 M Sodium citrate (Sigma, cat. no. S4641)
500 mM Sodium sulfite (Sigma, cat. no. S8018)

1 For 1 ml chromosome suspension, stain overnight with 5 µl of 1 mg/ml HO (final concentration 5 µg/ml), 5 µl of 10 mg/ml CA3 (final concentration 50 µg/ml) and 10 µl of 1 M MgSO_4_·7H_2_O (final concentration 10 mM). Then add 10 µl sodium citrate (final concentration 10 mM) and 50 µl of 500 mM sodium sulfite (final concentration 25 mM). Store in the dark at 4°C overnight before flow analysis and sorting.
  Sodium citrate and sodium sulfite have been used to improve flow karyotype resolution for most lymphoblastoid cell lines. The effect of both compounds on resolution can vary by cell line. Monitor the flow karyotype by reducing the concentration or excluding one or the other compound.
2 Proceed to Basic Protocol 4 or Basic Protocol 5.

## Basic Protocol 4 BIVARIATE FLOW KARYOTYPING ON A CELL ANALYZER

This protocol describes bivariate chromosome analysis on a cell analyzer after staining with DAPI and PI. This dye combination can be measured using a laser combination of 355 nm and 561 nm or 355 nm and 488 nm. The 5‐laser BDLSR Fortessa cell analyzer used in this example requires filter replacement on the 488‐nm blue laser optics configuration. The workflow described here is applicable to and compatible with other cell analyzer models.

### Materials

BD LSRFortessa cell analyzer equipped with the standard 5‐laser system (355 nm, 405 nm, 488 nm, 561 nm, and 640 nm)
Long pass LP 620 filter (optional)
Cell staining buffer (Biolegend, cat. no. 420201)
BD FACSDiva CS&T Research beads (BD, cat. no. 655051)
5‐ml BD Falcon tube (SLS, cat no. 352054)
20‐µm mesh filter (CellTrics, cat. no. 04‐004‐2325)

1 Briefly vortex the stained chromosome sample for 5 s.
2 Centrifuge the chromosome suspension at 201 × *g* for 3 min at 4°C. Filter supernatant through 20‐µm mesh into a 5‐ml BD Falcon tube. Store on ice.
3 Perform daily maintenance on the BD LSR Fortessa according to manufacturer instructions.
4 Perform an instrument performance check using BD CST beads. Vortex beads thoroughly for 5 s. Mix 2 drops of beads with 500 µl cell staining buffer in a 5‐ml Falcon tube. Verify that the cytometer performance check passed before proceeding to experiment set‐up.

### Setting up parameters and plots for chromosome analysis

**Figure 4:**
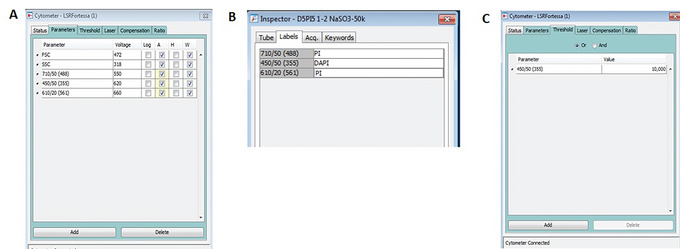
Chromosome analysis setup on the BD LSRFortessa cell analyzer. (**A**) Setting up the required parameters (**B**) Label the selected parameters (**C**) Setting the threshold.

5 Using the parameters specified below, set up the instrument for the chromosome analysis experiment as shown in Figure 4A.

**Figure 5:**
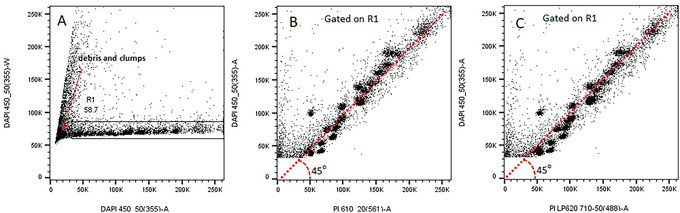
Data plots for chromosome analysis on a BDLSR Fortessa cell analyzer. (**A**) DAPI 450_50 (355)‐A versus DAPI 450_50 (355)‐W (**B**) DAPI 450_50 (355)‐A versus PI 610_20 (561)‐A (**C**) DAPI 450_50 (355)‐A versus PI LP620 710_50 (488)‐A.

6 Under Parameters Panel:
  - For DAPI detection, select 450/50 (355). Set signal detection to Area and Width.
  - For PI detection using the 561 nm laser, select 610/20 (561). Set signal detection to Area and Width.
  - For PI detection using the 488 nm laser, select 710/50 (488). Set signal detection to Area and Width. On the Octagon detection array for the 488 nm laser, replace the 685 DLP with a blank filter holder and swap the band‐pass filter BP 710/50 with a band‐pass filter BP 610/20 or a long‐pass filter LP 620 (optional).
  - Delete any unnecessary parameters.
7 Label the parameters as shown in Figure 4B.
8 In the Cytometer Settings menu, set the Threshold level at 10,000 for 450/50 (355), as shown in Figure 4C.
9 Create the dot plots as shown in Figure 5 for (A) DAPI 450_50 (355)‐A versus DAPI 450_50 (355)‐W, (B) DAPI 450_50 (355)‐A versus PI 610_20 (561)‐A, and (C) DAPI 450_50 (355)‐A versus PI LP620 710_50 (488)‐A.

10 In the Cytometer fluid control panel, adjust the RUN to a data rate of 1000 events per second.
11 In the Cytometer Settings menu, adjust the voltage for both detectors with the chromosome clusters displayed along a 45° angle as shown in the data plots (Fig. 5B and 5C).
12 Draw a region gate R1 on plot DAPI 450_50 (355)‐A versus DAPI 450_50 (355)‐W to gate out the debris and clumps (Fig. 5A).
13 In this example, both laser combinations (355 nm with 561 nm and 355 nm with 488 nm) are used simultaneously for chromosome analysis. Display gated events R1 on DAPI 450_50 (355)‐A versus PI 610_20 (561)‐A (Fig. 5B) and DAPI 450_50 (355)‐A versus PI LP620 710_50 (488)‐A (Fig. 5C).
14 Acquire and save 50,000 to 100,000 events.
15 Analyze and compare the collected chromosome data to the data obtained in Figure 17A. Verify if most chromosomes clusters can be identified. For further guidelines, refer to ‘Critical Parameters and Troubleshooting’.

## Basic Protocol 5 BIVARIATE FLOW KARYOTYPING ON A CELL SORTER

This protocol provides the procedure for bivariate chromosome analysis after staining with DAPI and PI on an Invitrogen Bigfoot spectral cell sorter. This dye combination can be measured using a laser combination of 355 nm with 561 nm or 355 nm with 488 nm. The workflow is applicable to and compatible with other cell sorters.

### Materials

Invitrogen Bigfoot spectral cell sorter equipped with 349 nm, 488 nm, or 561 nm lasers
70‐µm nozzle
Bigfoot Calibration Beads (Invitrogen, cat. no. PL00287)
20‐µm mesh filter (CellTrics, cat. no. 04‐004‐2325)
5‐ml BD Falcon tube (SLS, cat. no. 352054)
1.5‐ml Eppendorf tube, sterile, UV‐treated
Chromosome sheath buffer (see recipe)

1 Briefly vortex the stained chromosome sample for 5 s.
2 Centrifuge the chromosome suspension at 201 × *g* for 3 min at 4°C. Filter supernatant through 20‐µm mesh into a 5 ml tube. Store on ice.

### Setting up the cell sorter

3 Replace the sheath tank with chromosome sheath buffer.
4 Set the sheath pressure to 60 PSI.
5 Install a clean 70‐µm nozzle.
6 Start up the cell sorter as directed by the manufacturer.
7 Perform daily maintenance on the cell sorter as directed.
8 Perform instrument laser alignment and drop delay calibration using Bigfoot calibration beads according to manufacturer instructions. Verify that the sorter passed daily QC and the drop delay is established before proceeding to experimental setup.
  The Bigfoot cell sorter can perform both automated and manual laser alignment. Manual laser alignment is recommended for the chromosome application,. For other cell sorters, please use the recommended fluorescent beads and perform the daily QC laser check as directed by the manufacturer.

### Setting up parameters and plots for chromosome analysis

**Figure 6:**
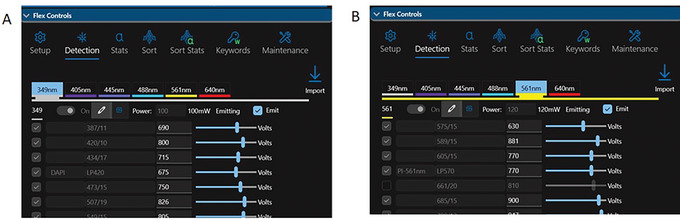
Chromosome analysis on an Invitrogen Bigfoot spectral cell sorter. (**A**) Setting up DAPI detection with the 349‐nm laser (**B**) Setting up PI detection with the 561‐nm laser.

9 Select ‘Quick Run’ and select the parameters used for the chromosome analysis experiment.
10 In Flex Controls, select ‘Detection’ and relabel the selected parameters (Fig. 6).
  For DAPI detection, within 349 nm parameters select band‐pass BP 455/14. Under ‘Optical Filters’, replace BP 455/14 with a long‐pass LP 420 filter.
  For PI detection, within 561 nm parameters select band‐pass BP 625/15. Under ‘Optical Filters’, replace BP 625/15 with a long‐pass LP 570 filter.
  These long pass filters are supplied by the instrument manufacturer with the instrument at purchase.
  (Optional) If using a 488‐nm laser for PI detection, select BP 615/24 in the 488 nm parameters.

**Figure 7:**
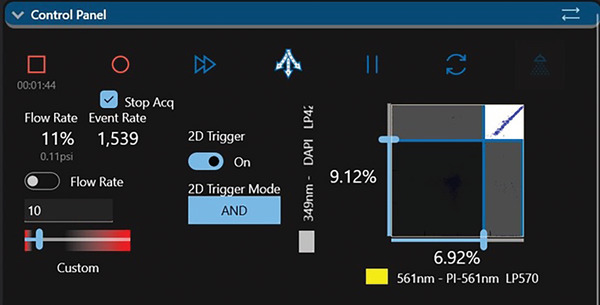
Set Trigger on parameters selected for 349 nm and 561 nm. Optimize the level to minimize background noise and debris.

11 In the Control Panel (Fig. 7), select 2D Trigger ‘ON’ and 2D Trigger mode ‘AND’. Optimize the level for both parameters selected for 349 nm and 561 nm or 488 nm (optional: if 488 nm laser is used for PI excitation).
  Bigfoot is equipped with a dual parameters trigger. If using other cell sorters, set the trigger value on a single parameter such as PI or DAPI fluorescence.

**Figure 8:**
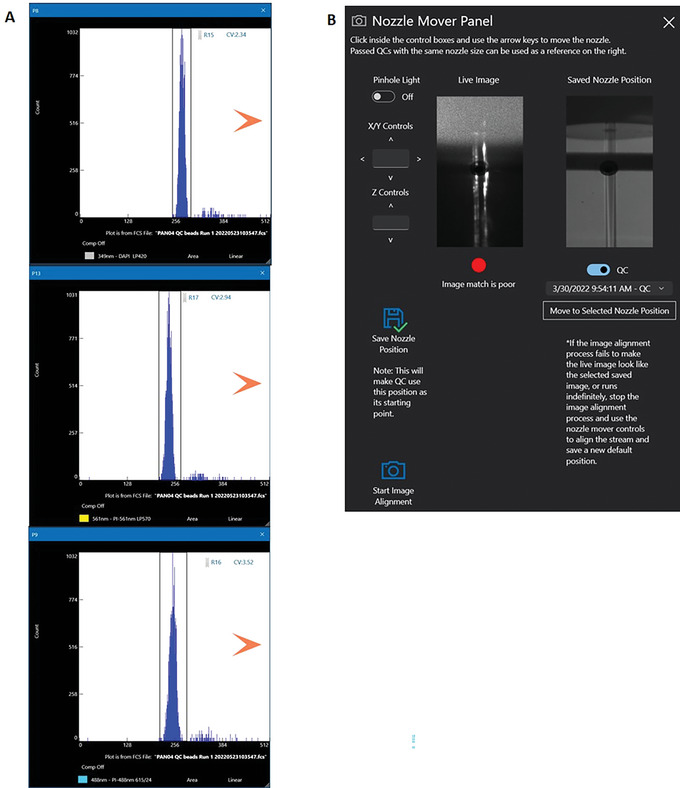
Histogram plot for parameters from 349‐, 561‐, and 488‐nm lasers. Set a region gate for each of the displayed histograms (**A**) Using the Nozzle Mover Panel control, align the lasers with minimum CVs for the DAPI and PI fluorescence channels (**B**).

12 Create a single parameter histogram plot for 349 nm‐DAPI LP420, 561 nm‐PI LP570, and 488 nm‐PI 614/24 (Fig. 8A).

### Manual laser alignment

13 Run a tube containing the Bigfoot calibration beads at a flow rate of 250 events/s.
14 Under ‘Flex Controls’, select ‘Nozzle Mover’ (Fig. 8B). On the Nozzle Move Panel align the optical light path with minimum coefficient of variation (CVs) for both DAPI and PI fluorescent channels.

### Setting up bivariate plots for chromosome analysis

**Figure 9:**
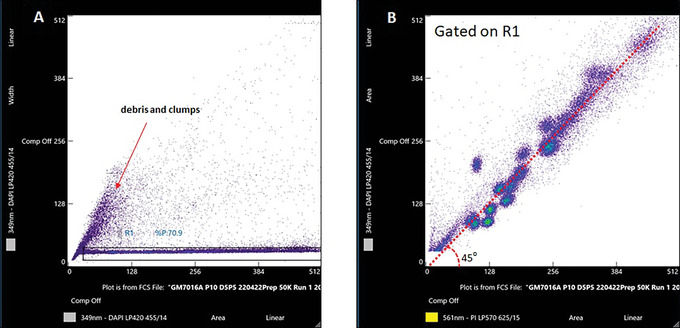
Data plots for chromosome analysis on an Invitrogen Bigfoot cell sorter. (**A**) 349 nm‐LP420 (Area‐Linear) versus 349 nm‐LP420 (Width‐Linear) (**B**) 349 nm‐LP420 (Area‐Linear) versus 561 nm‐PI LP570 (Area‐Linear).

15 Create the dot plots (Fig. 9) for (A) 349 nm‐DAPI LP420 (Area Linear) versus 349 nm‐DAPI LP420 (Width Linear) and (B) 349 nm‐DAPI LP420 Area Linear versus 561 nm‐PI LP570 Area Linear.

16 Optimize the RUN with flow rate 1000‐1500 events per second.
17 Draw a region gate on plot 349 nm‐DAPI LP420 (Area –Linear) versus 349 nm‐DAPI LP420 (Width‐Linear) to gate out the debris and clumps (Fig. 9A).
18 Display gated events in R1 on 349 nm‐DAPI LP420 (Area‐Linear) versus 561 nm‐PI LP570 (Area‐Linear). On ‘Detection’ (Fig. 6), adjust the voltage until the chromosome clusters are displayed along a 45° angle as shown in the data plot (Fig. 9B).
19 Acquire and save 50,000 to 100,000 events.
20 Analyze and compare the collected chromosome data to the data displayed in Figure 17B. Verify that most chromosome clusters can be identified. For further guidelines, refer to Critical Parameters and Troubleshooting.

### Sorting chromosomes

**Figure 10:**
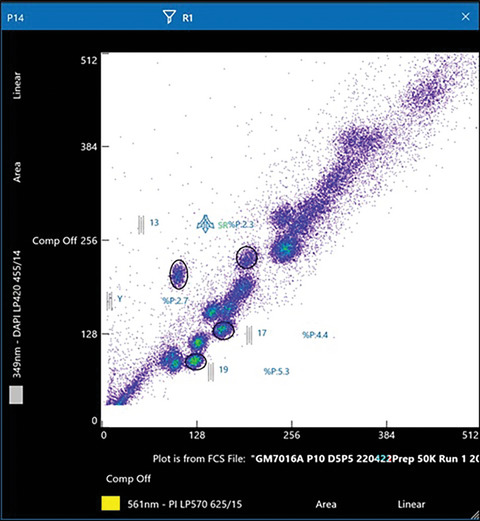
Draw a region gate around the chromosomes of interest for sorting. In this example, chromosomes Y, 13, 17, and 19 have been selected for sorting.

21 On the data plot, draw the region gate around the chromosome cluster of interest for sorting (Fig. 10).

**Figure 11:**
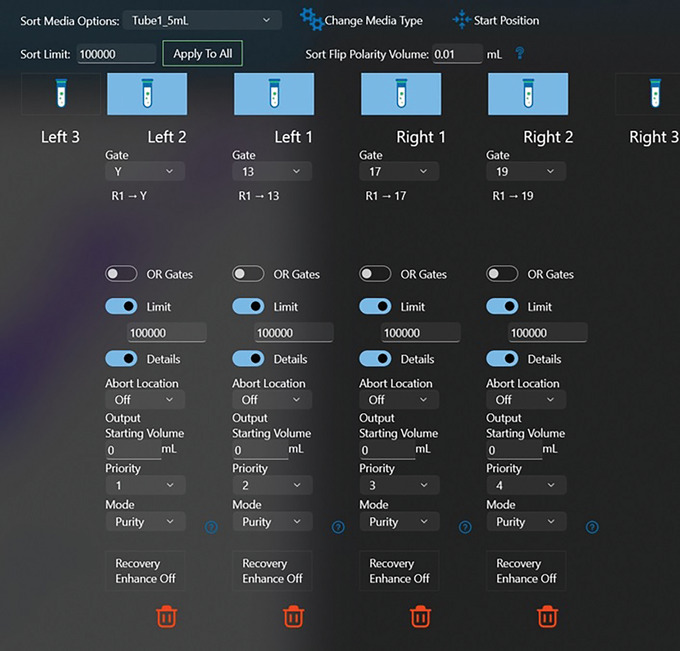
Set Sort Mode to Purity and the required sorted chromosome numbers per tube.

22 Within the Sort function, select sort media option ‘Tube1‐5ml’ for sorting into 1.5‐ml Eppendorf tubes. Calibrate and adjust the test sort stream to make sure the sort stream is aligned with the collection tube. Then set Sort Mode to ‘Purity’ and the required number of chromosomes to be sorted per Eppendorf tube (Fig. 11).

**Figure 12:**
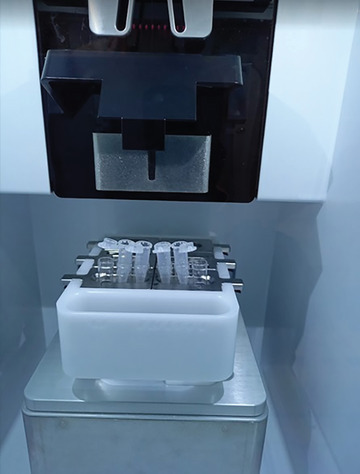
Position the empty 1.5‐ml Eppendorf tubes in the sort collection area.

23 Position the collection tube in the sorting collection area (Fig. 12).

24 Run the chromosome sample for a couple of minutes or until the chromosome clusters remain stable in their position as displayed in the data plot. Reposition the sort gate on the selected chromosome clusters if required.
25 Proceed with the sorting process.

## Basic Protocol 6 PURIFICATION OF FLOW‐SORTED CHROMOSOMES

This protocol provides the procedure for handling the chromosome material after sorting. In general, sorted chromosomes come in batches of approximately 250,000 copies per 1.5‐ml Eppendorf tube. The chromosome purification method described here is suitable for downstream processes such as PCR, sequencing, microarray, or other genomics assays.

### Materials

20 mg/ml Proteinase K (Invitrogen, cat. no. 25530‐015) (see recipe)
SARC/EDTA (see recipe)
20 mg/ml PMSF (Sigma, cat. no. P7626) (see recipe)
5 M NaCl (see recipe)
Absolute ethanol (VWR, cat. no. 20821.321)
Pellet Paint NF Co‐Precipitant (Sigma, cat. no. 70748)
Tris‐EDTA buffer (Sigma, cat. no. 93283)
Micro‐centrifuge (Eppendorf or equivalent)

1 Store the flow‐sorted chromosomes at 4°C.
2 Handle the Eppendorf tubes containing flow‐sorted material gently. Each tube should contain 250,000 chromosome copies in a 300‐µl volume.
3 Add 15 µl SARC/EDTA and 2.5 µl proteinase K. Incubate overnight at 42°C.
  The amounts of reagents required will vary according to the quantity of sorted sample.
4 Dilute 20 mg/ml PMSF to 4 mg/ml with absolute ethanol. Add 2 µl to the sorted DNA tubes. Incubate 40 min at room temperature.
5 Add 10 µl of 5 M NaCl (final 0.3 M including sheath buffer), 2 µl pellet paint, and 770 µl absolute ethanol. Mix gently by inversion. Store overnight at −20°C.
  The sorted chromosomes can be stored at −20°C at this stage until needed.
6 Pellet the sorted chromosomes at 15,700 × *g* for 15 min at room temperature.
7 Remove most of the supernatant with a P1000 pipette tip. Remove the remaining solution with a P200 pipette tip without disturbing the pellet.
8 Add 1 ml of 70% (v/v) ethanol without disturbing the pellet.
9 Centrifuge again for 7 min at 15,700 × *g*, room temperature. Remove the supernatant as in step 6.
10 Repeat 70% ethanol (v/v) wash one more time and allow the pellets to air‐dry at RT for 10 min.
11 Resuspend the pellet in 100 µl sterile Tris‐EDTA buffer.
12 Proceed to genomics assays, i.e., sequencing or microarray experiments.

## REAGENTS AND SOLUTIONS

Use sterile distilled water for all preparations. Use appropriate PPE when working with acids and bases.

### Chromomycin A3 (CA3), 10 mg/ml

Dissolve 10 mg CA3 in 1 ml absolute ethanol. Store in the dark at −20°C for up to 1 year.

### Chromosome sheath buffer (20×)

Mix the reagents to achieve the following final concentrations.
Prepare in a sterile 2‐L bottle.
100 mM NaCl (233.76 g)
1 mM EDTA (400 ml of 0.1 M stock)
10 mM Tris·HCl (400 ml of 1 M stock, pH 7.4)
Bring the volume to 2 L with sterile distilled water. Store at room temperature for up to 1 year.

### Chromosome sheath buffer (1×)

Prepare two sterile 2‐L media bottles.
Add 100 ml of 20× chromosome sheath buffer to each bottle and bring the volume to 2 L with sterile distilled water. Autoclave and store at room temperature for up to 1 year.

### DAPI, 1 mg/ml

Dissolve 1 mg DAPI (Sigma, D9542) in 1 ml sterile distilled water. Store in the dark at 4°C for up to 1 year.

### Dithiothreitol (DTT), 300 mM

Dissolve 1 g DTT (Gibco, cat. no. 15508‐013) in 21.6 ml sterile distilled water. Aliquot and store at −20°C for up to 1 year.

### EDTA, 0.5 M

Mix 9.31 g EDTA in 45 ml sterile distilled water. Dissolve with a minimum amount of 5 M NaOH (about 5 ml). Aliquot and store at room temperature for up to 1 year.

### Hoechst 33258 (HO), 1 mg/ml

Dissolve 1 mg HO in 1 ml sterile distilled water. Store in the dark at 4°C for up to 1 year.

### Hypotonic solution

Mix the reagents to achieve the following final concentrations.
Prepare in a 50‐ml Falcon tube.
75 mM KCl (3.75 ml of 1 M stock)
10 mM MgSO4.7H_2_O (500 µl of 1 M stock)
0.2 mM Spermine (25 µl of 0.4 M stock)
0.5 mM Spermidine (25 µl of 1 M stock)
Bring the volume to 50 ml with sterile distilled and adjust the pH to 8.0 using 0.25 M NaOH. Check the pH using pH indicator paper (Fisherbrand, 10333501). Store on ice. Prepare fresh on the day of chromosome preparation.
*CAUTION*: NaOH is an irritant; wear appropriate PPE when handling.
*CRITICAL*: Adjust pH to 8.0 before use.

### KCl, 1 M

Dissolve 3.72 g KCl (Sigma, cat. no. P9541) in 50 ml sterile distilled water. Aliquot and store at 4°C for up to 1 year.

### MgSO_4_·7H_2_O, 1M

Dissolve 2.47 g MgSO_4_·7H_2_O (Sigma, cat. no. M2773) in 10 ml sterile distilled water. Aliquot and store at 4°C for up to 1 year.

### NaCl, 5 M

Dissolve 5.85 g NaCl (Sigma, cat. no. S9888) in 20 ml sterile distilled water. Aliquot and store at room temperature for up to 1 year.

### PMSF, 20 mg/ml

Dissolve 250 mg PMSF (Sigma, cat. no. P7626) in 12.5 ml absolute ethanol to give a final concentration of 20 mg/ml. Aliquot and store at 4°C for up to 6 months.

### Polyamine isolation buffer (10× PAB)

Mix the reagents to achieve the following final concentrations.
Prepare in a 50‐ml Falcon tube.
800 mM KCl (add 2.98 g KCl in tube)
5 mM EGTA (2.5 ml 100 mM stock)
20 mM EDTA (2.0 ml 500 mM stock)
150 mM Tris (7.5 ml 1 M stock)
Bring the volume to 50 ml with sterile distilled water. Mix well and store at 4°C for up to 6 months.

### Polyamine isolation buffer (1× PAB)

Mix the reagents to achieve the following final concentrations.
Prepare in a 50‐ml Falcon tube.
Add 5 ml of 10× PAB and bring volume to 30 ml with sterile distilled water.
Then add the following
0.5 mM spermidine (25 µl of 1 M stock)
0.2 mM spermine (25 µl of 0.4 M stock)
3 mM Dithiothreitol (500 µl of 300 mM stock)
0.25% (v/v) Triton X‐100 (125 µl stock)
Bring the volume to 50 ml with sterile distilled water. Mix well using a rotator for 30 min and adjust the pH to 7.50 using 0.5 M NaOH. Check the pH using pH indicator paper (Fisherbrand, 10333501). Filter the buffer through a 0.22‐µm syringe filter. Store at 4°C for up to a month.
*CRITICAL*: Adjust the pH to 7.50 before use as pH can influence the stability and physical structure of the chromosomes.

### Propidium iodide, 1 mg/ml (PI)

Dissolve 1 mg PI (Sigma, cat. no. P4170) in 1 ml sterile distilled water. Store in the dark at 4°C for up to 1 year.

### Proteinase K, 20 mg/ml

Dissolved 100 mg Proteinase‐K (Invitrogen, cat. no. 25530015) in 5 ml sterile distilled water. Aliquot and store at −20°C for up to 1 year.

### SARC/EDTA, 10×

Dissolve 1 g sodium lauroyl sarcosine (Sigma, cat. no. L5125) in 5 ml sterile distilled water and 5 ml of 0.5 M EDTA. Store at room temperature for up to 1 year.

### Sodium citrate, 1 M

Dissolve 5.88 g sodium citrate (Sigma, cat. no. S4641) in 20 ml sterile distilled water. Aliquot and store at −20°C for up to 1 year.

### Sodium sulfite, 500 mM

Dissolve 1.26 g sodium sulfite (Sigma, cat. no. S8018) in 20 ml sterile distilled water. Aliquot and store at −20°C for up to 1 year.

### Spermine, 0.4 M

Dissolve 1 g spermine (Sigma, cat. no. S2876) in 7.18 ml sterile distilled water. Aliquot and store at −20°C for up to 1 year.

### Spermidine, 1 M

Dissolve 1 g spermidine (Sigma, cat. no. S2501) in 3.93 ml sterile distilled water. Aliquot and store at −20°C for up to 1 year.

### Turck's stain

Mix the reagents to achieve the following final concentration.
0.01% (w/v) gentian violet (Sigma, cat. no. 49144) in 1% (v/v) glacial acetic acid (Sigma, cat. no. A6283). Store at 4°C for up to 1 year.

*CAUTION*: Glacial acetic acid is flammable and can cause severe burns. Handle safely in a fume hood.

## COMMENTARY

### Background Information

Conventionally, high‐powered water‐cooled lasers have been required to produce the excitation needed to generate well‐resolved bivariate chromosome data by flow cytometry (Ng, 2010). A well‐resolved flow karyotype is crucial for subsequent chromosome isolation and purification by the cell sorter. However, the installation of a flow cytometer equipped with high‐powered lasers often involves additional plumbing work, as well as additional floor space to service the lasers. The expense of this intricate configuration has therefore restricted bivariate analysis of chromosomes to only a few laboratories. In this protocol, we demonstrate that flow karyotypes with well‐resolved chromosome peaks can be acquired using a combination of DAPI and PI on flow cytometers equipped with typical lasers and optics. The technique for chromosome preparation is simple and the analysis of stained chromosomes can be easily carried out in most laboratories who own a flow cytometer equipped with either a blue 488 nm or a yellow‐green 561 nm and a UV 355 nm laser.

### Critical Parameters and Troubleshooting

All possible issues that could arise during the chromosome preparation, analysis, and sorting process are listed below. It is important to check the quality of the chromosome preparation before analysis and sorting. The flow karyotypes displayed below are based on conventional dye staining with HO and CA3. Data were acquired from a decommissioned cell sorter, Mo‐Flo, equipped with two high‐powered, water‐cooled lasers. Similar effects are applicable to chromosomes stained with DAPI and PI.

#### Cell culture and metaphase harvest

**Figure 13:**
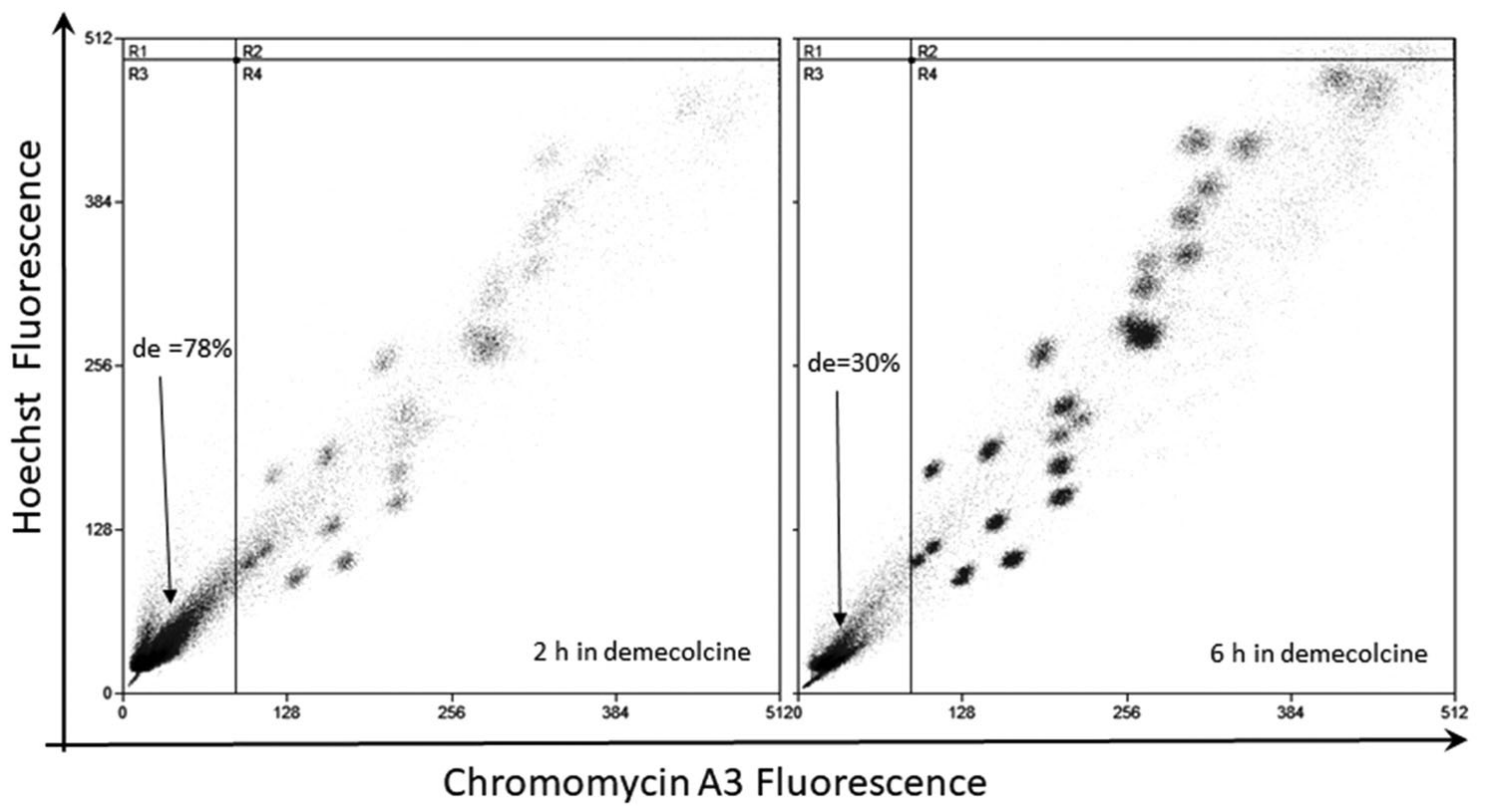
Bivariate flow karyotype plot of chromosomes from a human lymphoblastoid cell line. The cells were treated with 0.1 µg/ml demecolcine for 2 hr and 6 hr. Chromosomes were stained using the conventional dye combination of HO and CA3. The percentage of debris (de) generated during the chromosome isolation process was calculated by drawing a quadrant on the debris region and is shown in each panel. DNA containing debris is located at the lower left hand corner of the plots.

**Figure 14:**
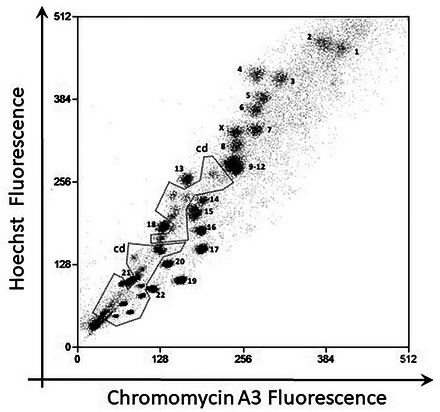
Bivariate flow karyotype of chromosomes from a human lymphoblastoid cell line. The cells were treated with 0.1 µg/ml colcemid for more than 8 hr. Chromosomes were stained using the conventional dye combination of HO and CA3. The normal chromosomes are labelled numerically. Chromatids (cd), which contain half of the DNA content of each of the corresponding chromosomes, can be observed at the lower end within the dotted line.

In general, we culture suspension cell lines using growth medium containing phenol red. Over time, the medium will turn yellow, which is an indication that the cells need sub‐culturing. We remove half of the old culture medium without disturbing the cell clusters and sub‐culture the near‐confluent cells at a ratio of 1:1 into two T‐75 cm^2^ culture flasks. For adherent cell lines, the cells are sub‐cultured when they reach approximately 75% confluence into two to four T‐150 cm^2^ culture flasks, depending on the cell growth rate. Following subculture, the cells are normally grown for 24 hr before treatment with 0.1 μg/ml colcemid. We treat the suspension and adherent cell lines with colcemid for 4‐6 hr and 6‐24 hr, respectively. The optimal conditions required for mitotic cell arrest varies among the cell lines. Optimization of the mitotic treatment time and dosage is recommended when working with different cell types. If a cell culture shows few mitotic cells, the cause is most likely insufficient incubation time with the mitotic inhibitors. This suboptimal condition often results in a chromosome prep that contains a high fraction of interphase cells, which contributes to the formation of debris and DNA fragments and has an adverse impact on flow karyotype resolution (Fig. 13). The yield of mitotic cells usually increases with extended treatment time. However, chromatid formation has been observed in cell lines that have been treated with colcemid for an extended period (Fig. 14). The presence of chromatids can impair the purity of the flow‐sorted chromosomes. The risk of chromatid contamination can be minimized by reducing the incubation time of the mitotic inhibitor (Telenius et al., 1993). We have also found that some cell lines are more susceptible to cell death upon treatment with colcemid. To overcome this issue, we recommend reducing the dosage of colcemid or replacing it with another mitotic inhibitor.

#### Chromosome preparation

In this protocol, the harvested colcemid treated cells are treated with hypotonic solution for about 15 min at room temperature. The swollen cells are monitored directly under the microscope by staining a small aliquot of the treated cells with Turck's stain or PI (Fig. 2A and 2B). With proper swelling, the cells are observed to increase in cellular volume and the chromosomes spread apart within the plasma membrane. The swollen cells are pelleted and drained dry on absorbent paper before being treated with a modified polyamine isolation buffer containing the detergent Triton X‐100. After detergent treatment, the cells are physically disrupted and the chromosomes released by vortexing or syringing. The chromosome suspension is then assessed for single intact chromosomes under a fluorescence microscope by staining a small aliquot of the suspension with PI (Fig. 3). With adequate swelling and vortexing, the chromosomes can be easily separated from one another and should appear as singly intact chromosomes in suspension. If the chromosome preparation contains chromosome clumps or bundles, disperse them by passing the suspension several times through a 22.5‐gauge needle on a 5 ml syringe.

#### Chromosome staining

**Figure 15:**
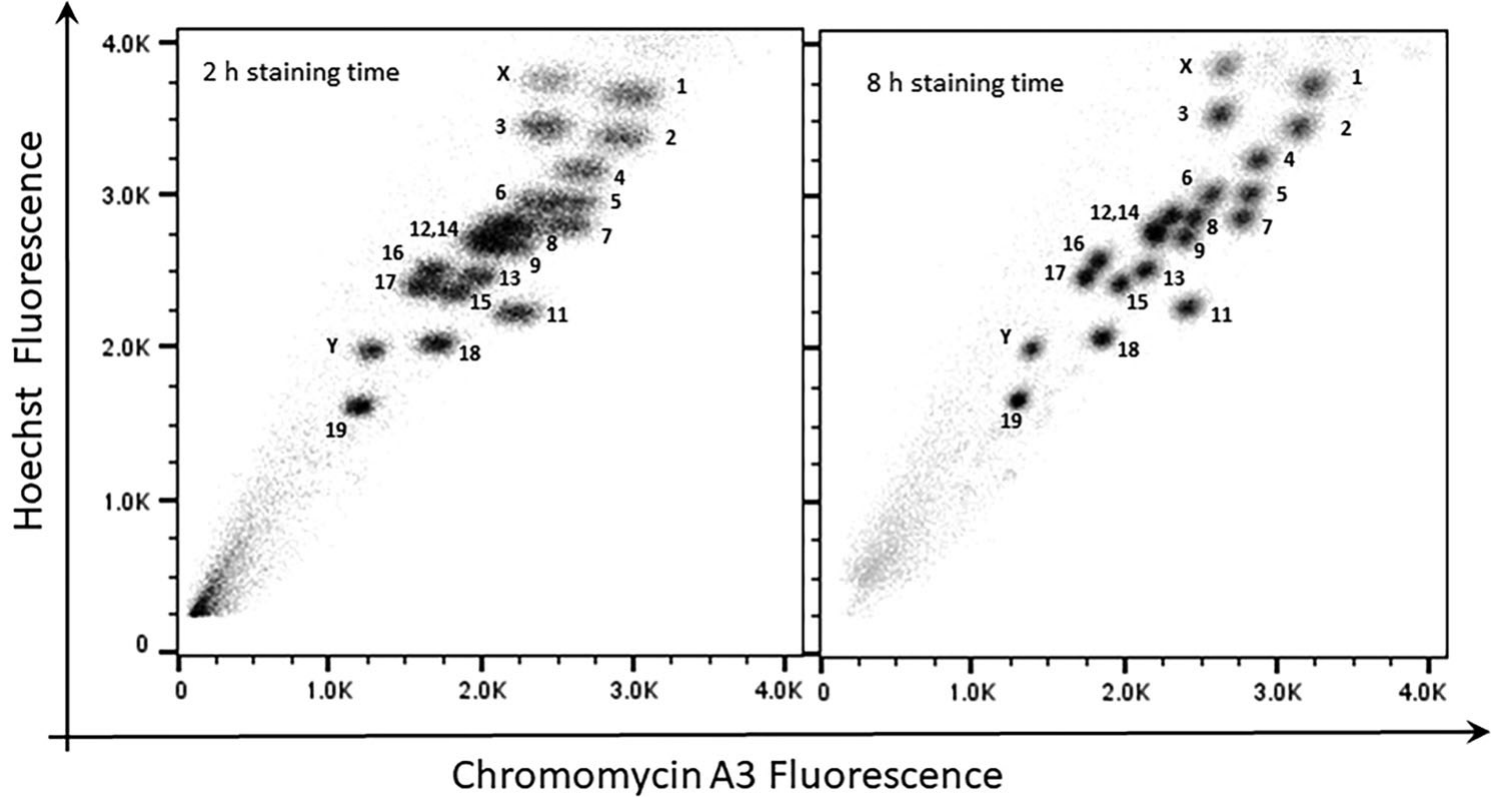
Bivariate flow karyotype of chromosomes from c57/BL6 LPS‐stimulated B lymphocyte mouse cell culture. Chromosomes were stained with HO and CA3 for 2 hr and 8 hr before analysis on a flow cytometer.

The degree of chromosome resolution is heavily dependent on the kinetics and interaction of the fluorochromes with the DNA (Langlois, 1979; Latt, Sahar, Eisenhard, and Juergens, 1980; van den Engh, Trask, and Gray, 1986). For optimum staining, sufficient time is required to allow the fluorochromes to equilibrate with the DNA. We found that extending the staining time beyond 2 hr improved the resolution of the flow karyotype (Fig. 15). A number of mouse chromosomes normally seen as a single multi‐chromosome cluster became more clearly resolved into multiple clusters after staining for 8 hr. For best results, we recommend staining the chromosomes overnight at 4°C in the dark.

In general, we add sodium sulfite and sodium citrate to the stained chromosome suspension and incubate overnight at 4°C to improve the resolution of the flow karyotype (van den Engh, Trask, Lansdorp, and Gray, 1988). However, the effect of both chemical compounds on the resolution of the flow karyotype varies between cell lines. Therefore, the use of these compounds must be monitored. In some cell lines, the flow karyotype resolution will deteriorate if both compounds are used. Therefore, we recommend reducing the concentration or excluding one or the other compound for these cell lines.

#### Flow cytometer optics

Different models of flow cytometer are available commercially. This protocol is suitable for most conventional flow cytometers that are equipped with the usual solid‐state lasers and a standard optical setup. In this protocol, we used a benchtop cell analyzer BDLSR Fortessa and a Bigfoot cell sorter. Both instruments are equipped with the standard lasers and optics as provided by the manufacturer. The fluorochrome for DAPI is excited with a UV laser (349 nm to 355 nm) and PI is excited with a 488‐ or 561‐nm laser. In a conventional flow cytometer, cell analyzers typically have a lower laser power setting built into the optic system; an attribute of its analytical feature when compared to a cell sorter. Nonetheless, flow karyotypes with good resolution can be obtained from cell analyzers with chromosomes stained using a combination of DAPI and PI (Fig. 17A).

Analysis of stained chromosomes can be performed using the standard optics supplied by the instrument manufacturer. In general, DAPI fluorescence can be measured with a band pass filter in the range of 400 and 490 nm (e.g., BP 450/50) or a long pass filter in the range >400 nm (e.g., LP 420). PI fluorescence can be measured with a band pass filter in the range of 600 and 640 nm (e.g., BP 610/20) or a long pass filter in the range >570 nm (e.g., LP 570, LP600 or LP620). The determining factor in your choice of optics is how well resolved the chromosomes are and the data resolution of the flow karyotype. We recommend optimizing fluorescence detection by using different detection filters, especially when working with cell lines that present a chromosome distribution with little or subtle differences in their DNA content and base composition.

#### Instrument set up and quality control

We normally set up the cell sorter for flow karyotyping using a 70‐µm nozzle with sheath pressure at 60 psi and the drop drive frequency between 90 and 99 kHz. The optical light path of the flow cytometer is aligned using fluorescence particles or beads with minimum peak CV for all fluorescence channels. Flow karyotypes with good data resolution can be achieved by ensuring the instrument is properly aligned and has passed performance quality checks prior to analysis and sorting. On the Bigfoot cell sorter, the lasers can be aligned automatically or manually by using the proprietary Bigfoot calibration beads. On the bench‐top analyzer, this was performed using pre‐diluted BD CST beads (BD Cytometer Set up and Tracking Beads, BD Biosciences) on pre‐aligned lasers set up by the service engineer.

One of the main requirements of flow karyotyping is the ability of the instrument to distinguish the chromosomes from one another. A poorly maintained flow cytometer can impact the detection sensitivity and thus the overall data resolution. Therefore, it is essential to make sure that you have a clean and well‐maintained flow cytometer before performing any assay on the instrument. As part of a daily routine, we clean the nozzle for a couple of minutes with a sonicator and check for debris at the orifice under a light microscope. Occasionally, we use a compressed air duster to clear and force out any stubborn debris stuck at the nozzle orifice. It is also important to maintain the cleanliness of the fluidic line. In addition to the usual monthly cleaning recommended by the supplier, we normally clean the sample line with 1% (v/v) bleach followed by sterile water for at least half an hour before sample analysis and sorting.

#### Chromosome analysis

On the cell analyzer BDLSR Fortessa, we apply the threshold, or ‘trigger’, on the DAPI fluorescence channel as a way to exclude events that are not chromosomes. A total of 50,000 to 100,000 events were acquired at a data rate of 1000 events/s. We normally let the sample run for a couple of minutes until the chromosomes have settled and remain stable to allow equilibration of the fluorescent dyes in the fluidics line, before acquiring the chromosome events. To gauge the chromosome position, we draw region gates on a number of chromosomes of interest as a reference (Fig. 10). Occasionally, we acquire and refresh the data events for a couple of minutes to make sure the chromosomes are within the region gates before saving the events.

#### Chromosome sorting

On the Bigfoot cell sorter, we apply a combination of thresholds on both DAPI and PI fluorescence (Fig. 7) to exclude events that are not chromosomes. During chromosome sorting, we optimized the threshold to the lowest level with minimal effect on sort purity. Before performing the actual chromosome sort, a quick sort is done by sorting several chromosomes together within a large region gate and then reanalyzing the post‐sorted chromosomes for purity. The threshold is then adjusted accordingly with respect to the post‐sort purity. The chromosome samples can be analyzed and sorted between 4000 and 10,000 events/s depending on their concentration.

The quantity of chromosomes to be flow sorted is dependent on the experiments to be performed after sorting. We normally flow sort 500 chromosomes per chromosome type into PCR tubes containing 33 µl sterile UV‐treated water (Rabbitts et al., 1995). The amplified products can be used for chromosome painting assays (Carter et al., 1992). For large‐scale sequencing experiments, we recommend sorting at least 300 ng of DNA material per chromosome type. The amount of DNA material required per chromosome type can be computed using the information detailed in Table 1. The flow‐sorted chromosomes are then processed as described in Basic Protocol 6.

**Table 1:** Sizes of Chromosomes, an Estimate of the Number of Each Chromosome Needed to Yield 10 ng of DNA and an Approximate Amount of DNA (ng) from 250,000 Copies of Flow‐Sorted Chromosomes

Chromosome	Size (Mb)	Number of chromosomes to yield 10 ng DNA	DNA per 250,000 chromosomes (ng)
1	247	19,501	128.2
2	243	19,904	125.6
3	200	23,901	104.6
4	191	25,202	99.2
5	181	26,205	95.4
6	171	27,996	89.3
7	159	30,193	82.8
8	146	33,113	75.5
9	140	35,014	71.4
10	135	35,511	70.4
11	134	35,613	70.2
12	132	36,075	69.3
13	114	44,326	56.4
14	106	46,816	53.4
15	100	48,170	51.9
16	89	51,760	48.3
17	79	56,689	44.1
18	76	59,524	42.0
19	64	72,464	34.5
20	62	76,453	32.7
21	47	100,806	24.8
22	50	92,593	27.0
X	155	31,606	79.1
Y	58	85,911	29.1

Chromosome sizes according to Ensembl.

Number of chromosomes for 10 ng of DNA from CYDAC measurements (Mayall et al., 1984).

### Understanding Results

**Figure 16:**
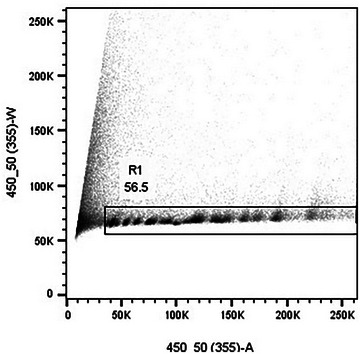
Gating out clumps and debris on DAPI fluorescence versus pulse width.

**Figure 17:**
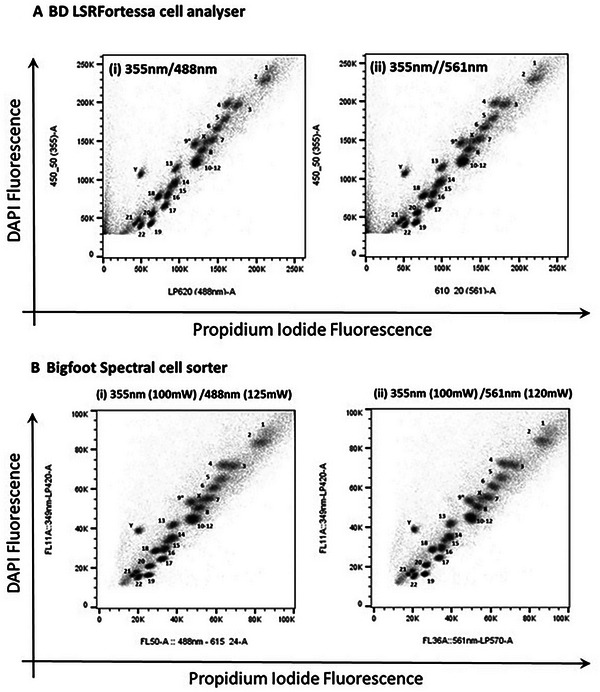
Bivariate flow karyotypes of chromosomes from human lymphoblastoid cell line GM7016A. Chromosomes were stained with DAPI and PI and acquired using conventional flow cytometers: (**A**) BD LSR Fortessa cell analyzer and (**B**) Invitrogen Bigfoot cell sorter with AT‐specific stain fluorescence on the Y‐axis, using laser combinations of 355 nm with 488 nm and 355 nm with 561 nm.

The protocols presented here describe the steps required to isolate chromosomes from mammalian cell lines using a modified polyamine isolation buffer (Ng, 2006). The isolated chromosomes are stained using a new fluorescent dye combination of DAPI and PI, which allows the stained chromosomes to be analyzed using flow cytometers equipped with standard lasers and optics (Ng et al., 2019). A total of 50,000 to 100,000 events were acquired and displayed on a bivariate plot of DAPI versus PI after gating out clumps and debris on DAPI fluorescence versus pulse width (Fig. 16). The bivariate flow karyotype of a normal male human cell line stained with DAPI and PI is shown in Figure 17. The data was obtained from flow cytometers available in the flow cytometry core facility. In this protocol, a bench top cell analyzer BD LSRFortessa and a cell sorter Bigfoot were used. With this dye combination, 24 chromosome clusters were revealed with the exception of the chromosome 10‐12 cluster. Chromosomes identified on the plots were numerically labelled with reference to the ISCN (International system for Human Cytogenetic Nomenclature) standard.

### Time Considerations

The process of harvesting cells for metaphase chromosomes followed by staining should take 2 days. The chromosomes are stained overnight before analysis and sorting. Flow cytometry analysis will take 10 to 30 min per sample, depending on the concentration.

### Author Contributions

**Bee Ling Ng**: Conceptualization, data curation, formal analysis, funding acquisition, investigation, methodology, project administration, resources, software, validation, visualization, original draft writing.

### Conflict of Interest

The author has no conflict of interest.

## Data Availability

The data that supports the findings of this study are available in this article.
